# The ridge-to-reef approach on Cicia Island, Fiji

**DOI:** 10.1007/s13280-021-01669-w

**Published:** 2022-01-11

**Authors:** Elodie Fache, Simonne Pauwels

**Affiliations:** 1 SENS, IRD, CIRAD, Univ Paul Valery Montpellier 3, Univ Montpellier, Montpellier, France; 2 Aix-Marseille Université, CNRS, EHESS - CREDO UMR 7308, Labex Corail, Marseille, France

**Keywords:** Environmental anthropology, Fiji, Integrated land-sea management, Marine protected areas, Organic agriculture, South Pacific

## Abstract

Many Pacific countries and territories embrace an officially recognized ‘ridge-to-reef’ approach to environmental management. This is the case of Fiji, where the Lau Seascape Strategy 2018–2030, led by Conservation International, aims for integrated natural resource management across 335 895 km^2^. This area includes Cicia Island, which deserves particular attention since, years before the design of the Lau Seascape Strategy, its population developed its own informal ridge-to-reef scheme, involving a combination of certified organic agriculture and locally managed marine closures. Based on 1 month of ethnographic fieldwork, this paper presents this scheme and highlights local perception and conceptualization of its positive effects on both the land and the sea. These reflect the *iTaukei* (Indigenous Fijian) concept of *vanua*, which intrinsically connects the health of the land, the sea, and their (human and non-human) dwellers, while stressing the importance of addressing land-sea processes and management efforts beyond an ecological perspective, i.e. through an engagement with the iTaukei relational ontology.

## Introduction

In the South Pacific region, many coastal communities embrace officially recognized ridge-to-reef (or R2R) approaches to environmental management. This is well illustrated by the GEF (Global Environment Facility) Pacific Ridge to Reef Programme that involves the United Nations Development Programme, the United Nations Food and Agriculture Organization, and the United Nations Environment Programme as implementing agencies. As part of this multi-agency initiative, 14 Pacific Island countries aim to “maintain and enhance [their] ecosystem goods and services (provisioning, regulating, supporting and cultural) through integrated approaches to land, water, forest, biodiversity and coastal resource management that contribute to poverty reduction, sustainable livelihoods and climate resilience”.^1^ In Fiji, ridge-to-reef approaches have been developed as part of ecosystem-based management efforts (Giffin et al. 2019), and their implementation has been scaled-up through the concept of seascape^2^ (Jupiter et al. 2012). In particular, the *Lau Seascape Strategy 2018–2030* (LSS),^3^ led by the Fijian branch of the non-governmental organization Conservation International (CI), is described as “a guideline for ecosystem-based management of the large number of islands to the east of Fiji” (CI 2018, p. 37). It aims to build a coalition of stakeholders/partners (including customary chiefs and local communities, government agencies, civil society organizations, and private sector operators) to apply integrated natural resource management, “from ridge to reef to ocean”, in the Lau Province and its surroundings waters, i.e. across about 25% of Fiji’s exclusive economic zone (CI 2018).

Ridge-to-reef approaches aim to incorporate land-sea processes into endeavours to “foster coral reef resilience” (Delevaux et al. 2018, p. 2) and ensure the sustainability of reef fisheries. Indeed, while their impacts vary across time and space, land cover and land use affect coral reef health through key ecological processes (Carlson et al. 2019). These processes include the regulation of the amounts of freshwater and contaminants (such as sediments, nutrients, herbicides/pesticides and pathogens) that reach coral reefs through surface flow or submarine groundwater discharge (ibid.). Hence, “coral reefs downstream of land disturbance are often degraded by disease; low larval recruitment and survival; low rates of calcification and photosynthesis; and mortality from hypoxia, tissue degradation, and macroalgal competition” (Carlson et al. 2019, p. 1). Such land disturbance can be as obvious as extensive deforestation, but can also be far less manifest in the landscape and nonetheless rank among the most significant contributors to sediment delivery to reefs, such as unpaved/dirt roads acting as “an active source of sediment, a runoff amplifier, and a rapid conduit towards the ocean” (Oleson et al. 2017, p. 9). While coral reefs themselves are often negatively impacted by sediment and other suspended particulate matter, these can also have significant effects on reef fish, such as damage to gill tissue and structure as well as impaired visual and olfactory acuity and thus reduced ability to find suitable habitat and food (Bainbridge et al. 2018; Comeral-Raynal et al. 2019), hence a potential decrease in fish biomass (Delevaux et al. 2018; Delevaux and Stamoulis 2020). However, some fisheries (especially small pelagics) may benefit from anthropogenic nutrients, e.g. from fertilizer application and sewage runoff, as illustrated by the coastal Mediterranean fishery off the Nile River delta in Egypt, and possibly occurring in the vicinity of “some rapidly developing tropical countries, where nutrient concentrations in the coastal waters were previously very low” (Oczkowski et al. 2009, p. 1364). Ridge-to-reef approaches are highly significant on high islands, as those found in the South Pacific, since land-sea connectivity is peculiarly strong in such contexts (Delevaux et al. 2018). For instance, in this region, marine species are “uniquely sensitive to land-based change” as many of them are diadromous, i.e. regularly migrate between the sea and freshwater (Carlson et al. 2019, p. 3). Interestingly, ridge-to-reef studies tend to present corals – and the fisheries and marine life they support – as “the ‘downstream’ endpoints of land-use byproducts”, instead of highlighting that land-sea connectivity is bidirectional (Carlson et al. 2019, p. 12). In addition, despite valuable exceptions (e.g., Jupiter et al. 2017; Leenhardt et al. 2017), ridge-to-reef studies rarely engage in more-than-ecological appraisals of land-sea processes and management efforts, acknowledging in particular the views of local and Indigenous communities.

**Fig. 1:**
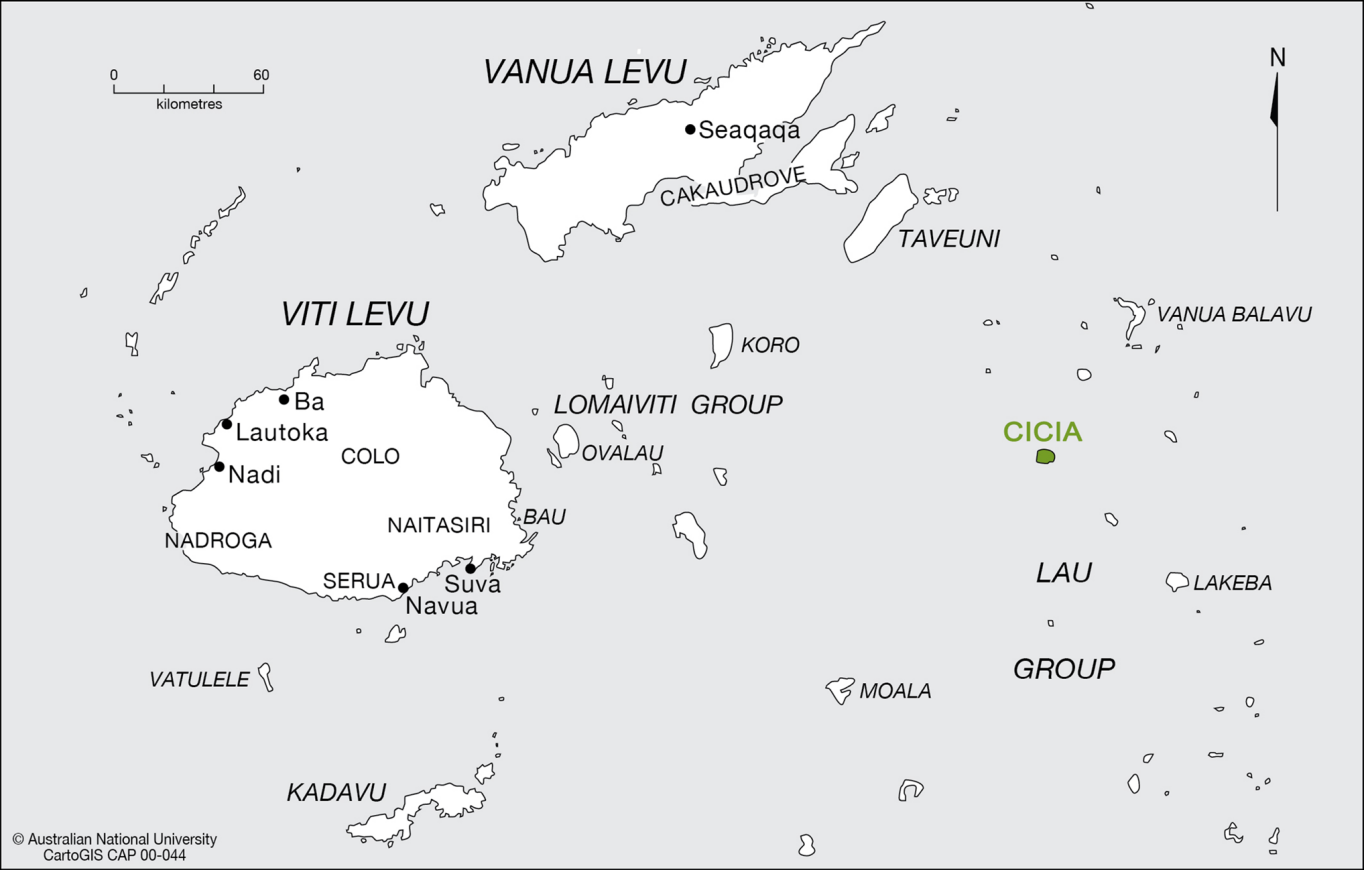
Map of Fiji highlighting Cicia Island in the Lau Province. Source: CartoGIS Services, College of Asia and the Pacific, The Australian National University, http://asiapacific.anu.edu.au/mapsonline/base-maps/fiji-main-islands, modified by the authors

This paper aims to fill this research gap, based on an ethnographic study we conducted in 2019 on Cicia (Fig. 1), one of the Fijian islands involved in the LSS. It will not focus on the latter, which was only nascent at the time of our fieldwork. It will rather focus on the informal ridge-to-reef scheme progressively developed and implemented by Cicia islanders several years before the CI-led design of this province-wide and multi-stakeholder strategy. This local scheme has involved the combination of an organic transition and certification of all farming activities at the island level with the creation of a network of periodically harvested marine closures (known as ‘*tabu* areas’). It is anchored in the *iTaukei* (Indigenous Fijian) concept of *vanua*, which refers to socio-ecological units as well as to a complex theoretical whole composed of socio-cosmic relationships (Chave-Dartoen 2016). The term *vanua* is often translated into English as ‘land’. Yet it encompasses a land-sea territory (including mountains, forests, rivers, beaches, reefs, etc.), the various beings (people, ancestors, spirits, fauna, flora, etc.) that dwell in this territory, and the socio-cultural settings and socio-political organization that shape this territory (Ravuvu 1983; Tuwere 2002; Nabobo-Baba 2006). It also has a “symbolic meaning” in addition to this “literal meaning” (Tuwere 2002, p. 33): the *vanua* is conceived as the main foundation of knowledge (Nabobo-Baba 2006); a source of life as well as a “social fact” holding life together and giving it meaning (Tuwere 2002, pp. 35–36); a source of identity and security, of belonging and confidence (Ravuvu 1983, p. 70); and “an extension of the concept of the self” (Ravuvu 1983, p. 70).

After a brief presentation of our study site and methods, we will present the informal ridge-to-reef scheme set up on Cicia Island (which may be used as a baseline for future studies of its articulations with, and changes caused by, the LSS as the latter unfolds in a post-pandemic world). Then we will highlight local perception and conceptualization of the positive effects of this scheme on both the land and the sea. This will allow us to emphasize that, in the frame of the concept of *vanua*, the land and the sea – as inseparable from their various dwellers – are considered in a relation of “mutual affecting and being affected” (Slaby et al. 2017). This perspective will thus contribute to a better understanding of “the connection between land use, water quality, fishing pressure, and the ecological condition of coral reef resources at whole-of-island scales in the Pacific Ocean” (Comeros-Raynal et al. 2019, p. 516), not through an ecological angle, but through an engagement with an Indigenous relational ontology (Poirier 2008; Fache and Pauwels 2020).

## Study site and methods

With a total land area of about 18 000 km^2^ but an exclusive economic zone covering more than 1.2 million km^2^, Fiji (or the Republic of the Fiji Islands) is a large ocean island state (Quirk and Hanich 2016). The archipelago includes more than 300 islands, of which about one third are inhabited, spread across 14 Provinces. Fiji’s total population stands around 900 000, living mainly in coastal areas. This is particularly the case in the Lau Province, in Fiji’s Eastern Division, where about 60 islands and islets, collectively known as the Lau Group, are scattered over 114 000 km^2^ of ocean. The Lau Group has been identified as “an area of national significance and high priority for marine protection” (Miller et al. 2018, p. 5). In this area indeed, the coral reefs are “generally healthy with high coral cover, diverse soft corals, and well developed deep-water coral communities” as well as “a high diversity of motile invertebrates” (Bruckner et al. 2016, p. 2). However, fish found on these reefs are unusually small, with few large fish remaining, which suggests that the fishing pressure might be too high (Bruckner et al. 2016, p. 3). A study conducted in 2018 on three islands of the Lau Group (Matuku, Moala, Totoya) estimated in particular that the two main groups of fish caught for food, lethrinids (emperor fish) and serranids (groupers), may, respectively, be nearing overexploitation and be already overexploited, hence a risk of collapse in these fisheries (Waqairatu-Waqainabete et al. 2019).

**Fig. 2:**
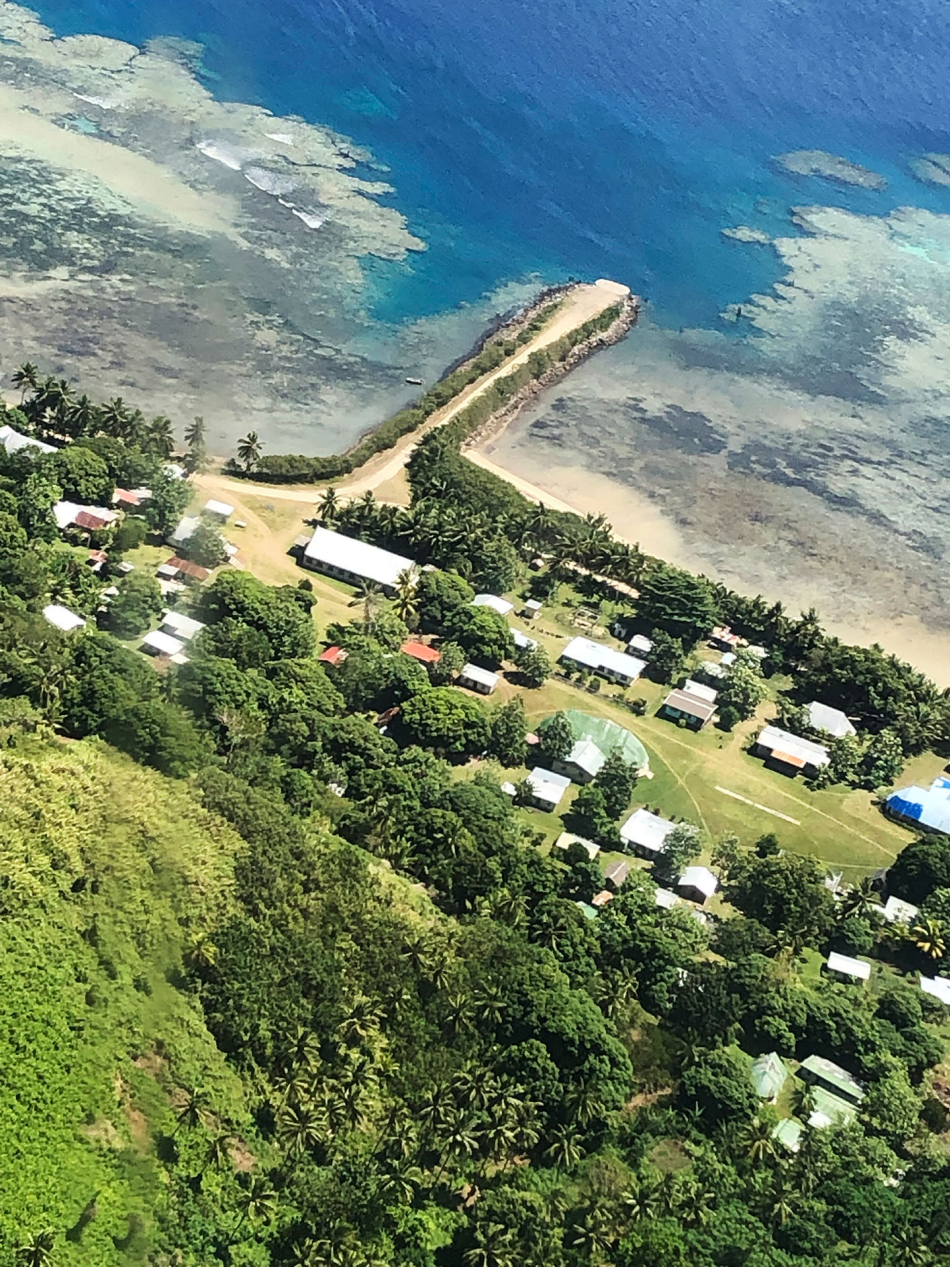
Aerial view of Tarukua village, Cicia Island, Fiji. Source: The authors, October 2019

The Lau Group includes Cicia, a small volcanic island (about 35 km^2^) surrounded by coral reefs (Fig. 2). Cicia forms a *tikina* or district that gathers five villages, all located in coastal areas, with a total of just over 1000 inhabitants (Table 1), mainly *iTaukei* Fijians. This district’s infrastructures include an unpaved road that circles the island, a jetty and an airstrip, as well as a health centre and a post office (in Tarukua), a high school (in Mabula) and three primary schools (in Tarukua, Mabula, Natokalau), and a Fisheries station and an ice plant officially opened in January 2019 (in Natokalau). There is currently no formal tourism operation on Cicia (see also Larsen 2021). Part of its population has a regular income, for instance as civil servants (teachers, health workers, fisheries or agriculture officers, etc.), as managers of a small grocery store, or through the sale of copra or virgin coconut oil.^4^ Yet most islanders mainly live from subsistence fishing (to which women actively contribute)^5^ and/or farming (i.e. cultivation of root crops such as yam, taro and cassava, vegetable crops, fruit trees, as well as breeding of a few pigs, lambs, goats, chickens…), while hoping to generate sustainable income from these activities in a near future. As Cicia is relatively distant from the capital city (Suva) and its markets, with only a weekly Twin Otter flight and a theoretically monthly – yet irregular – ferry, at present very limited amounts of fish caught and commodities produced on Cicia are exported (see also Larsen 2021).This article is based on qualitative data collected by the authors, with the crucial linguistic, social and practical assistance from Mere Veitayaki, during a four-week fieldwork period on Cicia, in September–October 2019. While we were based in Tarukua, we made several day trips to the four other villages of the island. Our immersion in Tarukua’s village life allowed for the participant observation of daily activities (such as fishing, cooking, production of virgin coconut oil, etc.), our involvement in community events (such as religious gatherings and collective meals on Sundays), informal discussions in these diverse contexts, and semi-structured interviews on the status and concept of ‘organic island’, local fishing practices and fisheries management efforts, as well as the socio-cultural context in which these are embedded. These interviews were conducted in Fijian and/or English, and recorded or not, depending on the preferences of each interlocutor and the particularities of each encounter. Our visits to other villages allowed for individual and group interviews with local leaders, elders and officers, as well as with both men and women fishers. In total, we carried out individual interviews with 30 adults on Cicia (and with some of them, we had several exchanges), as well as group interviews with women in four different villages (Tarukua, Naceva, Natokalau, Lomaji) and with men in Lomaji.^6^

**Table 1:** Cicia census provided by Cicia Health Centre in October 2019

Population/village	Males	Females	Total
Mabula	245	196	441
Lomati	104	101	205
Tarukua	96	63	159
Natokalau	69	82	151
Naceva	43	32	75
Total	557	474	1031

In addition to this fieldwork period on Cicia, this paper draws on the previous research experiences of the authors in Fiji. Elodie Fache carried out five months of fieldwork between 2016 and 2018 on Gau, Fiji’s fifth biggest island, located in the Lomaiviti Province (Fache and Breckwoldt 2018), as well as a short group fieldwork on Kadavu Island in 2019 (see Harding et al. in this special issue). Simonne Pauwels has conducted regular fieldwork periods on Lakeba Island in the Lau Province since 2005, as well as five months of fieldwork, between 2014 and 2017, in diverse places on Vanua Levu, Vanua Balavu, the Mamanuca, and the Yasawa Islands as part of an interdisciplinary project on the marine worm *Palola viridis* (Pauwels, in press). This paper also relies on a triangulation with secondary sources, in particular on ridge-to-reef management within and beyond the South Pacific and on the *iTaukei* concept of *vanua*.

## A local combination of marine closures and organic agriculture

### A network of five tabu areas

The customary fishing rights area – or *iqoliqoli* – of Cicia consists of the inshore waters, up to the outer limits of coral reefs, in which its *iTaukei* population has access, use and management rights, but that “fall within the territorial sea and therefore are under the sovereignty of the State” that retains the power to regulate or legislate resource uses (Sloan and Chand 2016, p. 79). Within this *iqoliqoli*, each village has established a periodically harvested marine closure or ‘*tabu* area’, located in its adjacent waters to allow for ongoing village-based surveillance. During our interviews, we have not been able to trace the exact date of establishment of each *tabu* area, but all were created several years – some of them more than 10 years – before the launch of the LSS. They aimed to reduce the fishing pressure within the *iqoliqoli*,^7^ which also faces other threats such as cyclones, rising sea temperatures, and outbreaks of the coral-eating crown-of-thorns (*Acanthaster spp.*) (Bruckner et al. 2016; MoF 2018, p. 5).

These closures can be temporarily opened after permission is given by the village’s customary chief, for community events only, such as funerals, collective fish drives (*yavirau*) during Christmas time, or visits from government officials. These openings were said to last from one day up to one week, during which finfish are the main or only target (with, in particular, a ban on giant clam harvesting explicitly mentioned in several villages^8^). Their frequency appeared to vary greatly from one village to another. Despite these differences, our interviewees all considered that their village’s *tabu* area had positive impacts on the island’s *iqoliqoli*. They highlighted that, within these closures, there was now an abundance of finfish, shellfish, seaweed, etc. and corals were recovering. These statements echo the findings of a study carried out in 2013 in ten islands of the Lau Province, including Cicia, highlighting that the sea cucumber abundance, while overall in severe decline, was significantly higher in *tabu* areas than in open/fished areas (Jupiter et al. 2013). However, while some of our interlocutors reckoned that the reintroduced giant clams were multiplying, others said that they disappeared due to tidal waves or poaching. Several interviewees also emphasised that marine resources were overflowing from these *tabu* areas to adjacent waters.

According to three interviewees, since the opening of the Fisheries station, discussions have emerged on the island about the future establishment of marine protected areas (MPAs) by way of regulations under Sect. 9 of Fiji’s Fisheries Act. These discussions generated ambivalent feelings since, we were told, so-called ‘gazetted MPAs’ would facilitate the surveillance of Cicia’s waters and thus help reduce poaching, but would not allow for periodical harvesting of marine resources as *tabu* areas do.

### A certified organic island

**Fig. 3:**
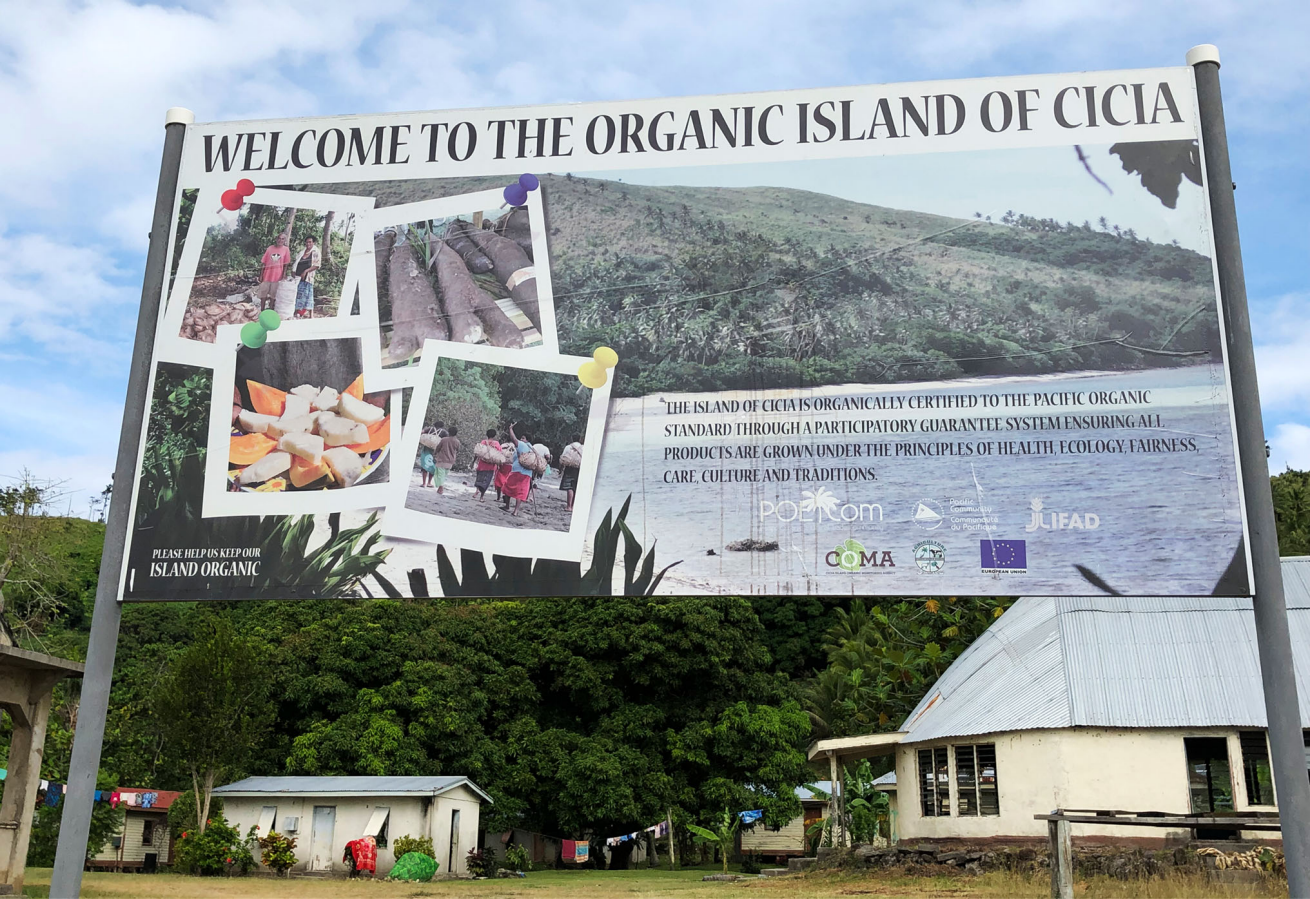
Board located at the entrance of Tarukua village, Cicia Island, Fiji. Source: The authors, October 2019

In 2006, the District Council (*Bose ni Tikina*) approved the idea of engaging in organic farming at the island level, and the use of chemicals (fertilizers, pesticides, weedicides) was therefore banned from Cicia. In 2013, the island was declared organic, not through a third-party certification process, but through a Participatory Guarantee System, based on the direct involvement of farmers in the organic guarantee process^9^ (Fig. 3). The implementation of this system involved the creation of Cicia Organic Monitoring Agency (COMA), gathering the island’s former and current agriculture officers, the District Council’s chairman, the district’s official representative (*Mata ni Tikina*), the headman of each village (*Turaga ni Koro*), and other representatives from each village. During our fieldwork period in 2019, the organic certification was granted to about 200 registered farmers, all village-based virgin coconut oil (VCO) groups, and Selavo Agro Business (a small-scale company owned and managed by the former agriculture officer and a local leader). While Cicia High School was not certified, its nearly 80 students – among which about 50 boarders – were also enthusiastically applying organic farming principles within the school ground.

These organic transition and certification processes were mainly set in motion and driven by the above-mentioned couple that runs Selavo Agro Business. Their leadership was based on their respective government-based and customary-based positions on the island, and their related focus on agriculture and on a transformed and transforming tradition (*cakacaka vakavanua*, literally working, acting, doing in the manner of the land; Toren 1999, p. 79). The organic turn was also influenced, among other circumstances and institutions, by: the historical production of copra on an estate covering a large part of Cicia, first leased by the Carpenters company, then by a cooperative named Cicia Plantation Company Limited; the increasing interest in higher-value coconut commodities, such as (virgin) coconut oil; the support to organic production provided by the Pacific Community^10^’s Land Resources Division based in Fiji; the creation of the Fiji Organic Association in 2007 and of the Pacific Organic and Ethical Trade Community (POETCom) in 2011; and last but not least, the Bible. In Fiji, where nearly all *iTaukei* people are Christians and the overall importance of Christianity has been well documented (e.g., Ryle 2010), some parts and specific verses of the Bible gain prominence in particular contexts (Tomlinson 2013), such as climate change (Fache and Fair 2020). The couple explained that the idea of applying the new concept of organic farming to Cicia came from a Bible study focused on the book of Daniel, which recommends fasting, i.e. consuming only food from one’s garden and water from streams (Daniel 1:11–17).

On Cicia, these organic farming principles include the use of alternatives to chemicals,^11^ and therefore the revival of ancient practices based on the idea that “our forefathers were smarter than us now” (interview with a member of COMA in Tarukua, 12 October 2019): using bird manure instead of chemical fertilizers, putting ashes or dried seagrass on cabbages and sand on yam mounds instead of chemical pesticides, etc. The current agriculture officer also encourages crop rotation on each plot and the careful washing of all seeds from outside the island before planting them. But above all, minimising or even ceasing the burning of vegetation on all land areas is locally seen as a crucial criteria of organic farming.^12^ This is in line with the general requirements for organic production defined in the Pacific Organic Standard, stating that slash and burn cultivation is now discouraged due to its impact on soil quality and biodiversity, and that land preparation by burning shall be restricted (SPC 2008, p. 12).

However, the global label ‘organic’ is not restricted to farming practices on Cicia, but also creatively used in relation to other activities (such as construction, fishing and seafaring; Larsen 2021) and related knowledge. For its inhabitants, this island’s organic certification “is not simply […] a process of commodifying products” to be exported, but allows for the local promotion of “an ‘organic lifestyle’”, which involves consuming non-imported, non-industrialized and non-monetarized food and other resources, such as timber (*ibid.*, p. 66). It also aims to revitalize customary behaviours and values, as illustrated below.

## Local perception and conceptualization of the effects of marine closures and organic agriculture

Ecological studies carried out in other contexts (e.g., Delevaux and Stamoulis 2020) suggest that such a combination of marine closures and organic agriculture, because it minimizes both fishing and land-based impacts, is an effective way to foster the resilience of coral reefs and associated fisheries. Indeed, without a regulation of land use and cover, marine closures are exposed to land-based pollution, and therefore, “not always capable of addressing human drivers that impact the benthic community of coral reefs upon which reef fishes depend” (Delevaux and Stamoulis 2020, p. 30). However, married with “vegetative land use”, marine closures “can improve cross-ecosystem outcomes” (Carslon et al. 2019, p. 4). In addition, terrestrial management is particularly beneficial “where marine closures may fall short”, such as beyond the boundaries of these marine closures (Delevaux and Stamoulis 2020, p. 30).

Our study did not assess this combination of marine closures and organic agriculture through an ecological lens, but endeavoured to understand its effects as perceived and conceptualized by the inhabitants of Cicia. Interestingly, our interviews highlighted that one of the main drivers of this island-level organic transition was the increasing depletion of marine resources: this changeover was considered as a solution for the recovering of marine resources. Some of our interviewees explicitly reckoned that, since the use of chemicals was banned, corals are growing well, seaweeds are abundant and green, and more seafood is available near the villages. It is also recognized that excessive burning on land leads to a depletion of fish in the sea, whereas the ban on burning results in an abundance of fish. Indeed, their view is that when the land is ‘dry’ (*dravu-i-siga*), instead of ‘green’ (*drokadroka*), the sea is ‘poor and unproductive’ (*dravudravua* or *drava*). As expressed by a religious leader: “if the land is rich, the sea too is rich” (interview in Tarukua, 7 October 2019), or by a customary leader: when the land is *‘sautu’*, the sea too is *‘sautu’* (interview in Tarukua, 2 October 2019). *Sautu* is usually translated as ‘peace and abundance’, with peace between people as well as between them and all other elements of the *vanua* (ancestors, spirits, fauna, flora, etc.) being the precondition of this status, and abundance of children, animals and plants being its most visible outcome.

The converse is also true: when the sea is plenty, the land is plenty too. This is best illustrated by the effects of the yearly arrival in Cicia’s waters of *balolo*, the marine worm *Palola viridis*, in October and/or November. Eight days after the full moon, *balolo* swirl from the bottom of the reef to the surface^13^ where the villagers scoop them with nets and bring them to the village where this much-desired delicacy is shared, wrapped in breadfruit leaves, cooked in an earth oven (*lovo*), and consumed. The days before *balolo* appear, the reef is said to be *drava*, ‘poor and unproductive’, as it is hard to get fish. Once the *balolo* show up, different fish species come to feast on this delicacy and spawn in turn, and people can therefore catch a lot of fish. *Balolo* do not only allow an increased availability of fish on the reef, but also an abundance in yam gardens. Indeed, the breadfruit leaves that are put in contact with *balolo* for their cooking are then placed on yam mounds in order to increase the flesh of yams and keep them healthy. This was and remains crucial, as in Fiji’s coastal areas yam is not only a staple food but also a pillar of *iTaukei* cultural practices.

The above-mentioned customary leader went a step further in the conceptualization of these land-sea interrelations in organic terms:

“The organic status of your inner being will also affect the organic status of your customary fishing rights area and land.” (interview in Tarukua, 2 October 2019; translated by Mere Veitayaki)

The status of people’s inner being is based on local values that, according to this leader (as well as other *iTaukei* Fijians we met on Cicia and in other parts of the country), now tend to be marginalised, first and foremost the sharing of subsistence resources with relatives, other community members, and sometimes people from other groups (e.g., groups having common borders or ties). This was (and to some extent remains) a pivotal *iTaukei* behaviour and value (Nabobo-Baba 2006, p. 88), ensuring that “the resources were efficiently used and that people looked after each other in times of need” (Veitayaki et al. 2014, p. 34). It was (and to some extent remains) also a core aspect of the “Fijian way”, conceived as “highly moral and ordered”, in opposition to “‘the European way’ or ‘the way in the manner of money’” regarded as “amoral and without order – an association of strangers” (Toren 1999, p. 27).

Indeed, more individualistic behaviours and values have progressively developed among *iTaukei* Fijians, including on Cicia, which some of our interlocutors illustrated through the example of changes in how *tugadra* (Bigeye scad, *Selar crumenophthalmus*, Carangidae family), a small pelagic species, is now fished in Tarukua. Formerly, when this culturally significant fish, supposed to be plentiful in April^14^ according to the traditional Fijian calendar (Veitayaki 2002, p. 396), was first spotted in Tarukua’s waters, the fisher(s) concerned used to call the whole village, whose most inhabitants engaged in a collective fishing party. Then, the catch was distributed between all households, including to the inhabitants who were not able to participate in this fishing. Nowadays however, fishers who notice *tugadra* no longer invite others to join them but catch them discreetly, and no longer share their take at the village level but consume it within their own household. As a consequence, interpreted most interviewees, this fish is now lost and rarely seen in Tarukua’s waters.^15^ The same kind of reasoning is made for the *balolo*: when it does not appear, this is often related to the fact that some people sold it instead of sharing it. More generally, this exemplifies how, from a local perspective, turning away from goodwill sharing and reciprocity has concrete consequences in terms of resource availability. Interestingly, in July 2021, while Fiji was under a Covid-19 lockdown, posts on Facebook announced that *tugadra* was spotted in Tarukua’s *tabu* area by two persons, who reported this information to the chief (Tui Tarukua), which was followed by a call of all villagers and a collective fishing party. The catch was then shared equally between all households. These posts, illustrated by many photos, generated a lot of comments, many of them presenting this event as ‘a blessing’, and one as a sign that both the sea and the land are *‘sautu’*.

## A *vanua* perspective on land-sea connectivity

Such local perception and conceptualization reflect that, in the frame of the *iTaukei* concept of *vanua*, “the world is one big entity and all things in it are associated”, with “life and its vitality depend[ing] on how all things relate and are affected by each other”; in particular, “life is complete and wholesome when all elements – air, wind, seas, rivers, plants, animals, fishes, people, the dead – are ‘synchronized’” (Nabobo-Baba 2006, p. 42). This term ‘synchronization’ is not defined by Unaisi Nabobo-Baba, but it seems to us that it echoes Anna Tsing’s idea of unexpected moments of harmony or coordination within polyphonic assemblages that gather human and other-than-human ways of being (Tsing 2015). We assume that the *iTaukei* term *sautu* might refer to the circumstances that result from such moments of harmony or coordination, when “life is complete and wholesome” (Nabobo-Baba 2006, p. 42).

In other words, all the human and other-than-human dwellers of a *vanua* are interconnected and have effects on one another. Therefore the abundance of a culturally significant marine species – such as *balolo* and *tugadra* – is seen as the sign that, in the terms of a village elder, “everything is on line”, “everything on the island is going well” (interview in Natokalau, 8 October 2019), with “everything” including the land-sea territory and its various inhabitants (*vanua*), spirituality/worship (*lotu*), kin relations (*veiwekani*), school and development. Conversely, when these marine species do not show up as scheduled in the traditional Fijian calendar (Veitayaki 2002), this is interpreted as a sign that the *vanua*’s dwellers are no longer in a relationship of mutual respect and reciprocity.

Customary chiefs play a crucial role in the maintenance of such a relationship of mutual respect and reciprocity and the search for plenitude of their *vanua*. We do not have the space here to present this role in detail, but it should be noted that the installed chief of a *vanua* is the only agency who can guarantee the harmony or coordination of the relationship between all the elements of this *vanua*, because he is the incarnation of the *mana*, i.e. the efficiency power, that flows from the ancestral spirits (*vu*) and spirit gods (*kalou vu*) to the whole *vanua* and that is essential to achieve a state of *sautu*. Without the agreement and support of the customary chiefs, none of the projects instigated by members of the *vanua* are possible. It was therefore crucial for CI to involve the forum of customary chiefs of the Lau Province (“the Masi ni Vanua o Lau”) in the design of the LSS, which also included the specific objective to “facilitate the installation of chiefs/leaders on each island” (CI 2018, p. 34).

A research pathway worthwhile investigating in future studies would be the (dis)connections between Cicia’s pre-existing scheme and this LSS that advocates an approach to environmental management “from ridge to reef *to ocean*”, and that articulates one of its seven specific strategies around the term *“bula sautu”* translated as “sustainable livelihoods” (CI 2018). While migrations between different islands of Fiji are at the core of the oral history of Cicia’s settlement that our interlocutors shared with us, while “interisland sociality does remain important to the everyday life of Lauan people” (Larsen 2021, p. 11), and while *balolo* and other marine species are thought to come inshore through reef passages, the open ocean was rarely mentioned in our discussions and interviews. While this might be partly related to the limited access the inhabitants of Cicia – including fishers – have to this space due to the lack of motorized boats, it seems to us that this reflects that Cicia is a *vanua* in its own right, working for its peace and abundance, and occasionally welcoming human and other-than-human beings coming in numbers from the open ocean (e.g. marine species during their spawning season and relatives during Christmas time). How does this fit with the LLS goals, for instance, to protect archipelagic waters and seamounts (CI 2018, pp. 47–48), and what role will Cicia islanders play to achieve these goals? In addition, how does the ambition of *“bula sautu”*/ “sustainable livelihoods” relate to the pursuit of peace and abundance (*sautu*) through a relationship of mutual respect and reciprocity between all dwellers of the *vanua*?

## Conclusion

**Fig. 4:**
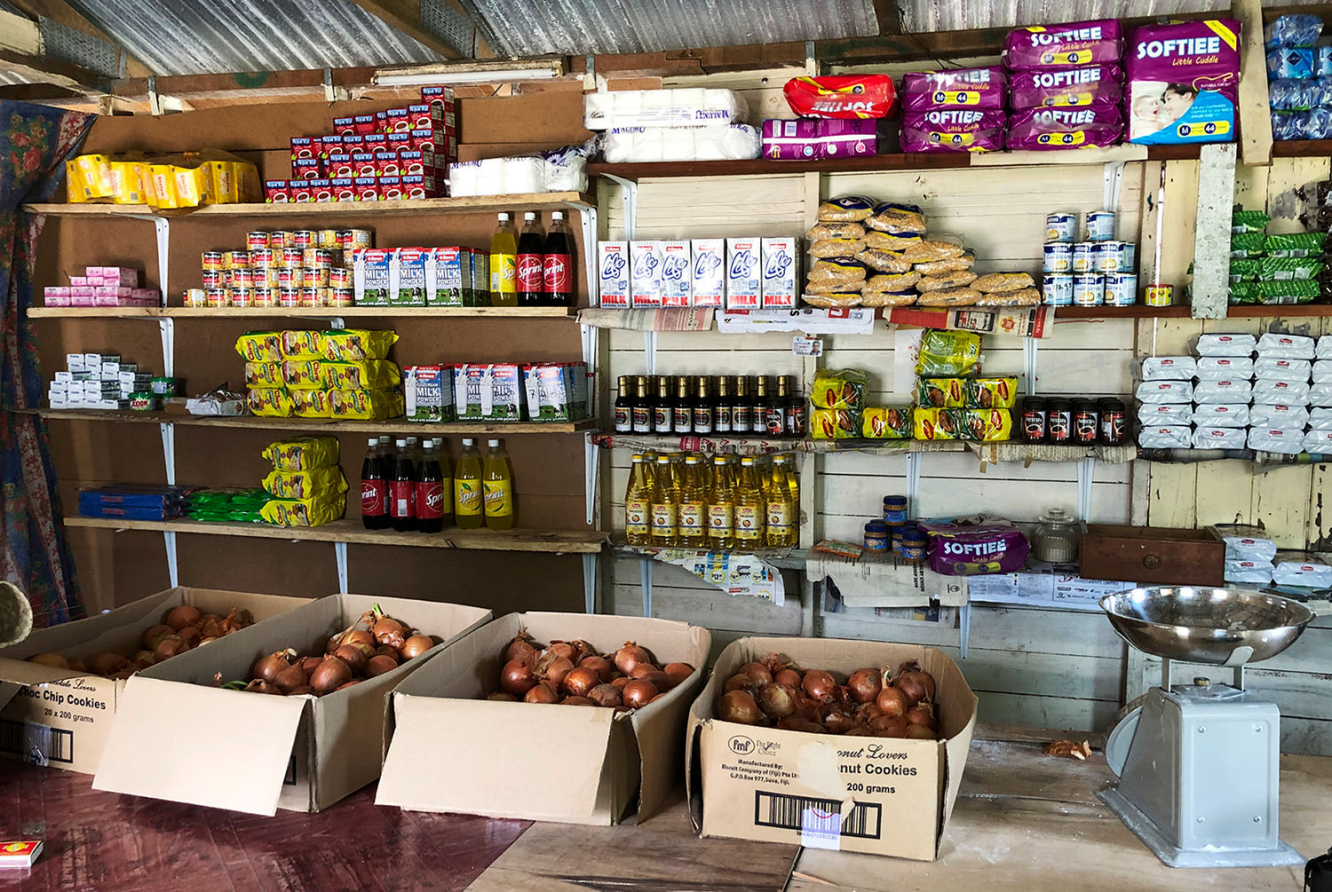
A local store/canteen. Source: The authors, October 2019

**Fig. 5:**
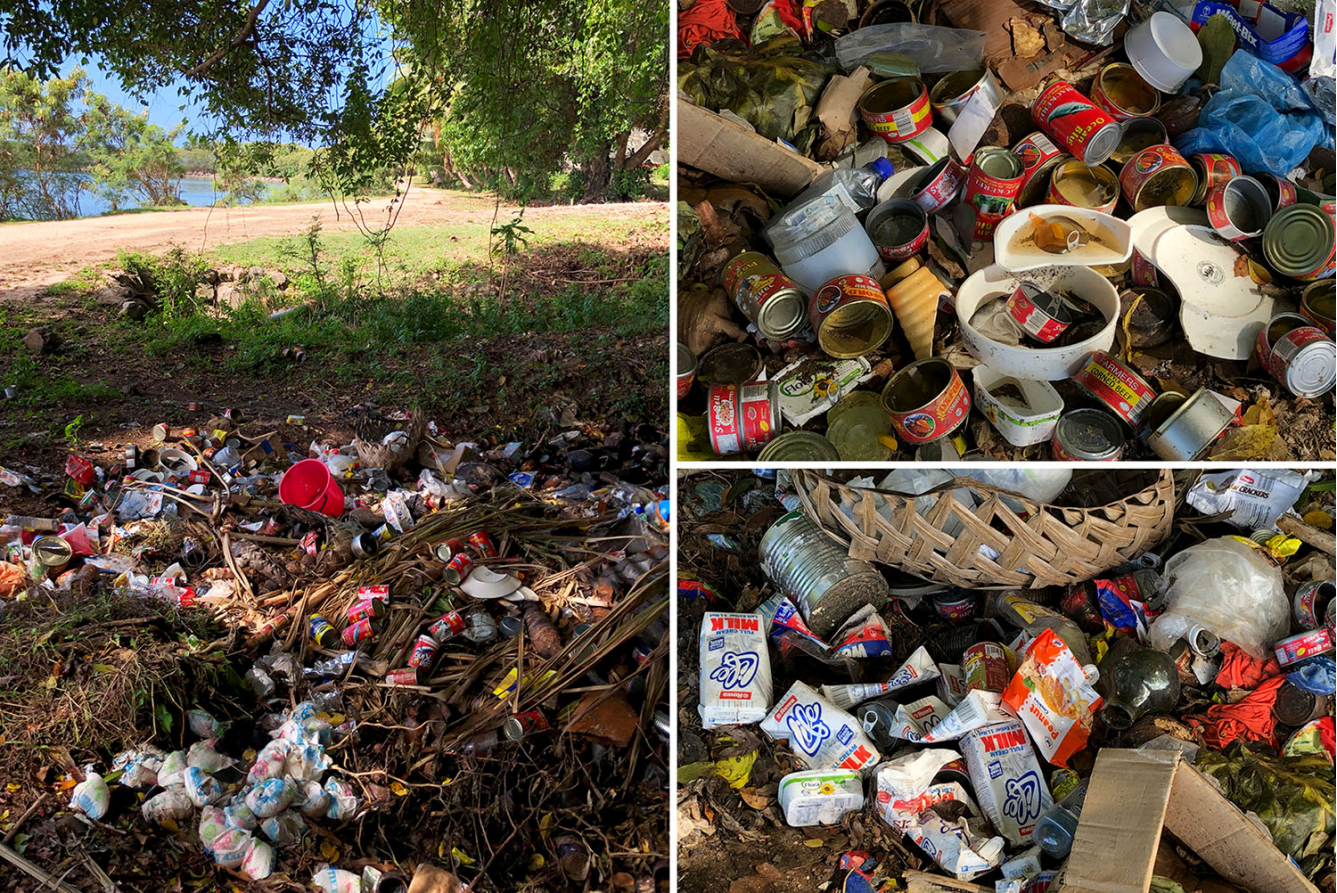
One of the two waste dumps in Tarukua village. Source: The authors, October 2019

During our fieldwork period in 2019, Cicia islanders pointed out that they were still facing several challenges in implementing their informal ridge-to-reef scheme. Poaching sometimes occurred in the *tabu* areas, while the overall fishing pressure remained high in the *iqoliqoli*. Some of our interlocutors thought that the ‘organic island’ concept was not yet fully understood by the population of Cicia, and that therefore the island was not 100% organic. For instance, they observed that unorganic/processed food was still sold and bought in the local grocery stores (wheat flour, soybean oil, canned fish, etc.; Fig. 4), not always by necessity but by taste, with their packaging accumulating in the waste dumps located at the land-sea interface (Fig. 5). In addition, more than five years after the organic certification, due to the island’s distance from the capital and irregular shipping services, no sustainable connection to markets had yet been established for its organic products, including virgin coconut oil which was rather distributed through personal connections with city dwellers. Yet one interviewee deemed this certification a “businessman move”; a statement to be understood in the light of the common idea among *iTaukei* Fijians that capitalist development is an immoral pursuit that challenges both the Christian and traditional relations, morals and obligations inherent in ‘the Fijian way’ (Toren 1999; Nolet 2018). We were also told that this ‘organic island’ concept could not solve all environmental issues (such as coastal erosion in Mabula), and had not (yet) involved any significant decrease in non-communicable diseases on the island (in particular diabetes, hypertension, strokes and heart problems).

However, this local experiment appears as significant at three different levels. First, it provides lessons that could inform the implementation of the LSS and, more generally, Fiji’s natural resource management policies. Second, it illustrates how people in the South Pacific region enact their “Oceanian sovereignty”, i.e. are asserting their right to make choices about the future of their land-sea territories and the ocean, through their fluid responses to the challenges confronting the wellbeing of the region across human and non-human domains (Bambridge et al. 2021). Third, in doing so, it provides a case study of Indigenous engagement in the design and implementation of marine governance and management arrangements, drawing on various concepts and registers of knowledge (Parsons et al. 2021).

**Fig. 6:**
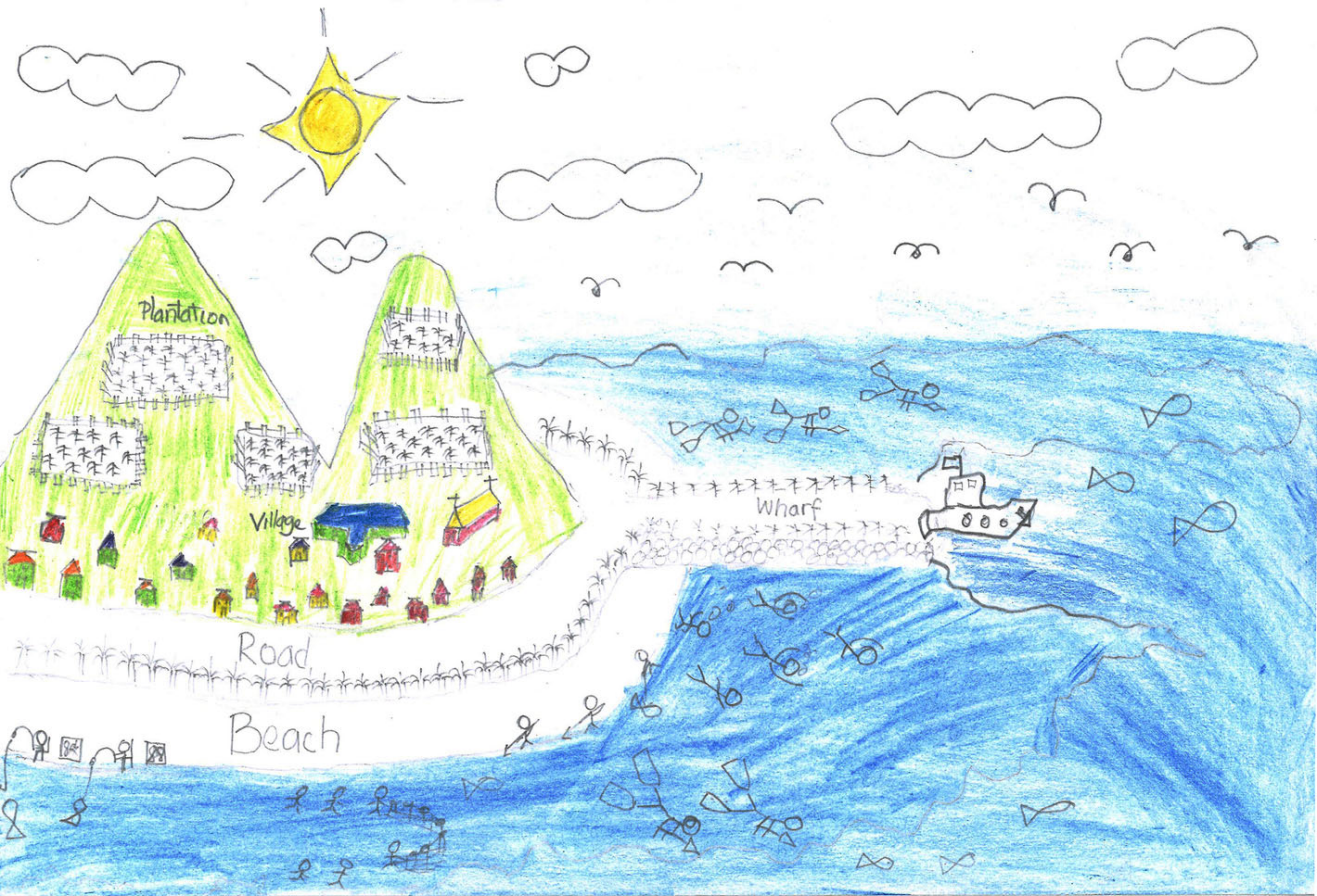
Inseparability between the land and the sea on Cicia Island, Fiji. Source: Drawing made by a 12-year old boy on Cicia in September 2019

Through this case study, this article brings more-than-ecological insights into land-sea connectivity in Pacific island settings, articulated around the *iTaukei* concept of *vanua* that conveys an inseparability between the land and the sea as inherent parts of an all-embracing entity (Fig. 6). Previous studies have shown that this land-sea inseparability manifests itself, for instance, in customary tenure and resource management encompassing “land-and-sea corporate estates” (Ruddle et al. 1992), in correlations between terrestrial and marine sources of food in the traditional Fijian calendar (Veitayaki 2002), and in the way *iTaukei* Fijians relate to specific fish and plant totems (Nabobo-Baba 2006, pp. 43 and 80). Here we draw attention, beyond this inseparability, to the interactive dynamics of relatedness and becoming between the land, the sea, and all their human and other-than-human dwellers.

## Footnotes

1 https://www.pacific-r2r.org/how-we-operate (Accessed on 25 May 2021).

2 Conservation International defines a seascape as “a network of marine protected areas, typically large, multiple-use marine areas, where governments, private organizations and other key stakeholders work together to conserve the diversity and abundance of marine life and promote human well-being” (https://www.conservation.org/projects/seascapes-program, Accessed on 2 July 2021).

3 https://static1.squarespace.com/static/5f6e6ced5f74dc06eded8ca2/t/5f7eb5adaad80539ea3edda2/1602139607711/Lau+Seascape+Strategic+Plan.pdf (Accessed on 2 July 2021).

4 That said, the sale of virgin coconut oil appears to provide only a small part of total household income on Cicia (Wara 2020).

5 The contributions of *iTaukei* women fishers to small-scale fisheries remain largely invisible, ignored and unrecognized, yet are crucial for household food security and livelihoods (Thomas et al. 2021).

6 In addition, we conducted short semi-structured interviews with 44 school children and the educational team of two primary schools as part of an ethnoecological methodology using drawings (see Fache et al. in this special issue), but this data is not analyzed in this paper.

7 Since our fieldwork period, the leaders of the Lau Province have introduced an overall ban on night spear fishing, which applies to Cicia and might also contribute to reduce the fishing pressure within its *iqoliqoli* (Fiji Sun, 10 June 2021, https://fijisun.com.fj/2021/06/10/lau-province-ban-night-spear-fishing-a-first-for-fiji/?fbclid=IwAR30q1IOyKNii7ZhfymA0s88C9tOTNtLf4lgo-WKqr3q7mrRsfGCBCBdDIo, Accessed on 22 October 2021).

8 Giant clams (*Tridacnidae spp.*) are listed under Appendix II of the Convention on International Trade in Endangered Species of Wild Fauna and Flora (**CITES**), implemented in Fiji through the Endangered and Protected Species Act. Since the early 1980s, Fiji’s Department/Ministry of Fisheries has been operating a hatchery on Makogai Island to restock reef areas with juvenile clams throughout the country. But as giant clams are a delicacy, some community members find it difficult to resist harvesting them.

9 https://lrd.spc.int/pacific-organic-standards/participatory-guarantee-systems-gps (Accessed on 31 May 2021).

10 The Pacific Community is an international development organisation owned and governed by Pacific countries and territories, as well as the principal scientific and technical organisation in this region (https://www.spc.int/, Accessed on 2 July 2021).

11 In 2016, on another rural island of Fiji’s Eastern Division, the authors witnessed the spraying of chemicals by subsistence farmers, and the local use of chemicals was confirmed in several interviews (with small-scale farmers, an agriculture officer, an NGO worker), for farming as well as for fishing purposes, e.g. to catch prawns in the river. However, on Cicia, it seems that the use of chemicals was not widespread prior to the organic transition (Larsen 2021, p. 76).

12 Interestingly, such a process of conversion from a shifting cultivation regime utilizing fire has historical precedents in the South Pacific region, for instance on Tikopia in the Solomon Islands where, however, it “took several hundreds of years and must have been built upon the accumulated trials and errors of many generations of gardeners” (Kirch 1997, p. 37).

13 Once or twice a year, the sexually immature male and female worms (atokes) transform into sexually mature worms by producing epitokes consisting of either sperm or eggs. These parts can grow to 18 cm. They detach from the atokes and move towards the water surface. They are what the *iTaukei* Fijians call *balolo*.

14 April is the beginning of the spawning season for this fish in Hawaii, where it is named *akule* and “shows site fidelity” (Weng and Sibert 1997, pp. 3 and 37). This primarily nocturnal fish is generally found in inshore waters and shallow reefs, where it travels in compact groups of hundreds of thousands of individuals (*ibid.*, pp. 7–8).

15 Based on existing literature, the decline of this small pelagic species might also be related to: overexploitation due to net fisheries (Smith-Vaniz et al. 2015) or to its status of both bycatch and baitfish in the increasing tuna fishery in Fiji (Muehlig-Hofmann 2007, p. 196); migration after events of soil erosion, as this fish prefers clean and clear waters (*ibid.*); or a reduction in nutrient loads discharged from the land into coastal waters (Gehrke 2007), e.g. due to the island’s organic turn.
